# Matrix and Backstage: Cellular Substrates for Viral Vaccines

**DOI:** 10.3390/v6041672

**Published:** 2014-04-11

**Authors:** Ingo Jordan, Volker Sandig

**Affiliations:** ProBioGen AG, Goethestr. 54, Berlin 13086, Germany; E-Mail: volker.sandig@probiogen.de

**Keywords:** vaccine production, host cells, vaccine substrate, continuous cell lines, primary cell culture

## Abstract

Vaccines are complex products that are manufactured in highly dynamic processes. Cellular substrates are one critical component that can have an enormous impact on reactogenicity of the final preparation, level of attenuation of a live virus, yield of infectious units or antigens, and cost per vaccine dose. Such parameters contribute to feasibility and affordability of vaccine programs both in industrialized countries and developing regions. This review summarizes the diversity of cellular substrates for propagation of viral vaccines from primary tissue explants and embryonated chicken eggs to designed continuous cell lines of human and avian origin.

## 1. Introduction

The ability to perform successive infectious cycles in cell culture was an important step for research on viral diseases and development of vaccines. Already in the late 1920s and 1930s, primary cell cultures were infected with vaccinia virus, poliovirus, and yellow fever virus to investigate and produce vaccines [1,2,3]. Experiments in 1929 also confirmed that an “increase of vaccine virus...did not occur in the absence of living cells” [1]. Such results are obvious today only because of the pioneering work at that time (which later, in 1954, resulted in the awarding of a Nobel Prize to John Enders, Thomas Weller, and Frederick Robbins for propagation of poliovirus in tissue culture).

One challenge was to differentiate virus propagation from mere survival of infectious particles in cultures with very low proliferation rates. In 1925, glycerinated testis of rabbits infected with vaccinia virus (VACV, *Orthopoxviridae*) were used as inoculum for cultures of testis from uninfected rabbits; titration of infectious units suggested an amplification of 51,000-fold over the course of 56 days [4]. This report favourably complements a publication in 1937 on the propagation of rabies virus in mouse brain cultures [5], that suggested that all previous reports on *ex vivo* amplification of rabies may have suffered from the ambiguity between true replication and mere rescue of input virus.

To provide more robust vaccine research options, and because of some disadvantages associated with manufacture in primary cells, other cell substrates have been developed. Today, the spectrum of substrates used for production of licensed and investigational vaccines ranges from primary cells (sometimes still within the animal) to designed continuous cell lines. Properties of these substrates with focus on historical aspects pertaining to human vaccines will be summarized in this review.

## 2. Finite Cell Cultures

Finite cell cultures are derived from primary cell preparations [6]. They are homogenized explants of tissues or complete animals and, consequently, a complex mixture of cells from different lineages. After the first passage, the culture constitutes a cell line. Depending on source and culture conditions, the cells usually stop proliferation within 50–70 cell doublings. At the time of its discovery this property, also called the Hayflick limit, came as a surprise [7,8] and is one disadvantage of finite cell cultures.

Different tissues from various animals were prepared for finite cell cultures from the 1910s to the 1960s for vaccine research. Technology based on embryonated chicken eggs, Indian rhesus monkeys, African green monkeys, and human primary cells was developed to a degree that large vaccine volumes were supported.

### 2.1. Primary Monkey Kidney Cells

Freshly prepared primary monkey kidney cells in Medium 199 were an important substrate for production of vaccines against infection with human adenoviruses (HAdV, *Adenoviridae*) and polioviruses (*Picornaviridae*). Adenoviruses are critical in the context of military operations, less so in the general population [9]. They can cause acute upper respiratory disease with the potential for severe epidemics especially in newly recruited military personnel [9,10]. The first vaccine against this febrile illness was a formalin-inactivated preparation that consisted of viruses of the most relevant serotypes 4 and 7 [9]. Thousands of recruits received this vaccine between 1955 and 1963.

Polio research was also heavily dependent on primary monkey kidney cells. Poliomyelitis was a severe and widespread global problem in the past century. Three vaccines were in development: a killed vaccine proposed by Salk [11] with virulent polioviruses as seed, and two live attenuated vaccines that were developed by Sabin [12] and by Koprowksi and Cox [13,14]. The ambitious and successful 1955 polio vaccine field trial was performed with the killed vaccine produced in minced monkey kidney cells suspended in chemically defined Medium 199 supplemented with calf serum [15,16].

However, the “fact that 1500 monkeys are needed in the production of every million doses of killed-virus vaccine has created great difficulties in the supply of monkeys” [17], and India, the main country of origin for the wild monkeys, was increasingly more reluctant to export the animals [18].

Although the production of live attenuated poliovirus also depended on primary, freshly prepared monkey cultures [19], it was argued that live vaccines are more efficient at inducing a protective immune response than their killed counterparts. Because fewer infectious units will be required for vaccination the burden on substrate availability may be lower [17]. The polarization of the political world during the cold war and in the polio research community may have contributed to the fact that Sabin’s live vaccine initially was predominantly used to control polio worldwide [20] whereas Salk's killed vaccine remained the preferred preparation in North America until 1962 [21]. Both vaccine types are associated with advantages and problems. The killed vaccine had a lower potency and there were instances where the complex procedure used to inactivate the virulent strain failed tragically [22,23]. In the live attenuated strains, a capacity for reversion to neurovirulent versions was demonstrated [24]. The risk that recipients of the live (and more potent) vaccine may shed revertants was difficult to quantify and balance against the high rates of wildtype poliovirus circulating in the population [17]. Today, there are no autochthonous poliomyelitis cases in most regions of the world and a killed vaccine is considered to be the best approach to control circulation of the remaining poliovirus serotypes in the few endemic areas [20]. But even with vastly improved production processes and shift to captive-bred monkeys as source [25], primary monkey kidney cells were not an option for vaccine manufacture anymore in the 1950s so that other cell substrates were investigated as an alternative (Table 1).

**Table 1:** Tissue cultures and cell lines used or intended for human vaccine virus propagation.

			killed	live
finite cultures	primary	monkey kidney	PV, HAdV	PV
		mouse brain	HNTV, JEV	
		hamster kidney	JEV	
		embryonated eggs	IAV, IBV	LAIV, YFV
		CEF	IAV, IBV, RABV, TBEV	MV, MuV MVA
	passaged	FRhL-2		RV
		WI-38		RUBV, HAdV
		MRC-5	HAV	PV, VZV
continuous	spontaneous	Vero	PV, JEV, RABV	RV,VACV
cell lines		MDCK	IAV, IBV	
		Sf9, Tn5	AcNPV	
		EB66		
	designed	HEK 293, PER.C6, CAP		
		AGE1.CR.pIX		

AcNPV, recombinant baculovirus for expression of heterologous antigens and virus like particles; HAV, hepatitis A virus; HNTV, Hantaan virus; IAV, IBV, influenza A and B viruses, respectively; JEV, Japanese encephalitis virus; LAIV, live attenuated influenza A or B viruses; MuV, mumps virus; MV, measles virus; MVA, modified vaccinia virus Ankara; PV, poliovirus; RABV, rabies virus; RUBV, rubella virus; RV, rotavirus; TBEV, tick-borne encephalitis virus; VACV, vaccinia virus; VZV, varicella-zoster virus; YFV, yellow fever virus; HAdV, human adenovirus. There are no licensed vaccines yet for EB66 and the designed cell lines.

Currently available vaccines produced on primary mammalian cells protect against hemorrhagic fever with renal syndrome and Japanese encephalitis (Hantavax, Korea Green Cross, and JE-VAX, Sanofi Pasteur/BIKEN, both propagated in mouse brain, and SA 14-14-2 JE, Chengdu Vaccine Plant, derived from primary hamster kidney cells).

### 2.2. Chicken Embryo Fibroblasts

The embryonated chicken egg is currently the most important source for primary cells for vaccine production. The embryo is extracted from the egg and enzymatically or mechanically homogenized. The resulting suspension contains the chicken embryo fibroblasts (CEFs) and is transferred to bioreactors for infection. Alternatively, virus is inoculated into the allantoic cavity to infect the embryos within the egg, taking care not to kill the embryo prematurely by mechanical manipulation. Primary chicken cells were already used in the 1920s for virological experiments. They were used in the 1940s and 1950s for propagation of mumps virus, poliovirus and rabies virus (and several veterinary vaccine viruses) and were also proposed as an alternative substrate to mammalian brain tissue-derived vaccines, because the latter carried the risk of inducing allergic encephalitis [26,27,28]. Primary chicken cells also provided substrates for generation of attenuated strains that are important today.

One such strain is yellow fever virus (YFV, *Flaviviridae*) 17D that is derived from Asibi, a highly virulent isolate obtained in 1927 from a Ghanian patient. In 1937, Theiler and coworkers attenuated the Asibi isolate by successive passages in primary chicken cells. An important variation was that brain and spinal cord were removed from the embryos prior to cell cultivation [2,29]. Between passage 89 and 114, 17D lost both its capacity for visceral infection and its neurovirulence for monkeys. Virulence of the related strain 17E, obtained via 240 passages in mouse embryonic tissue, did not change significantly compared to the parental Asibi isolate. This observation is consistent with the conclusion [29] that serial propagation of YFV in a non-mammalian substrate depleted for nervous tissue is the driving force behind this attenuation. Generation of the 17D strain was honored with a Nobel Prize in 1951, and today the vaccine against yellow fever still consists of the 17D strain but is produced in embryonated chicken eggs.

Another live vaccine attenuated by serial passaging on chicken-derived material is modified vaccinia virus Ankara (MVA; *Poxviridae*) [30]. The parental virus is the chorioallantois vaccinia virus Ankara (CVA), a smallpox vaccine maintained by alternating passages in the skin of calfs and donkeys at the Turkish Vaccine Institute in Ankara in the 1950s [31]. Starting in 1958 in Germany, an isolate of CVA was propagated serially in chicken embryo fibroblasts. At passage level 516, genetic stability and a host-restricted phenotype were confirmed in a plaque-purified isolate that received the name MVA to allow differentiation from other attenuated versions of vaccinia virus [31]. Characterized by several mutations and six scattered larger deletions [32,33], MVA can no longer replicate in human cells [34,35,36]. However, poxviral genes (and transgenes of recombinant viruses) are expressed very efficiently and induce a robust T-cell mediated immune response [37,38,39,40] that is not inhibited by preexisting immunity [41]. Because vaccinia viruses can be manipulated genetically by homologous recombination [37,42,43] and because of the excellent safety profile of MVA, this chicken cell-adapted virus strain is a highly promising candidate for vectored vaccine approaches [44,45,46,47,48]. Strain MVA-BN, a repeatedly plaque-purified strain derived out of MVA at passage 584, was approved as a vaccine against smallpox in adults in the European Union in 2013.

The eggs intended for use in vaccine production today are recommended to be derived from flocks of a well-defined regulatory status, referred to as Specific Pathogen Free (SPF). As described in Chapter 5.2.2 of the European Pharmacopoeia, SPF flocks should be protected against contact with non-SPF animals, including wild birds, rodents or insects. The risk of contamination is reduced by treatment of food and air, and personnel should shower and change into protective clothes before entering the controlled facility. Currently, SPF chicken flocks are screened for eighteen pathogens including avian adenoviruses, Marek’s disease virus, Newcastle disease virus and mycoplasma. No medication that may interfere with the sensitivity of detection of pathogens should be administered, and records of the general health of the flock should be maintained.

CEFs are used for production of vaccines against measles and mumps (for example, M-M-R II, Merck), tick borne encephalitis (FSME IMMUN, Baxter), and rabies (RabAvert, Novartis), embryonated eggs for production of vaccines against yellow fever (YF-VAX, Sanofi Pasteur), influenza (for example, INFLUVAC TC, Abbott and live attenuated viruses in fluMist, MedImmune), and smallpox (IMVANEX, Bavarian Nordic).

### 2.3. Diploid Cells

Other primary cell lines that are used in the production of current vaccines are the human diploid cells (HDCs) that were isolated in the 1960s, WI-38 [49] and MRC-5 [50]. WI-38 originates from a female human fetal lung approximately 13 weeks into pregnancy and MRC-5 from a male human fetal lung approximately 14 weeks into pregnancy. HDCs also suffer from the Hayflick limit but, as opposed to CEFs, multiple expansion passages of material obtained from well-characterized cryogenically preserved Master Cell Banks and Working Cell Banks allows the use of this valuable material in essentially closed systems.

MRC-5 and WI-38 are used for production of vaccines against rabies (for example, IMOVAX RABIES, Sanofi Pasteur), hepatitis A (HAVRIX, Glaxo Smith Kline and VAQTA, Merck), and varicella (ZOSTAVAX, Merck; VARILRIX, Glaxo Smith Kline).

WI-38 is also used for production of the military vaccine against acute respiratory disease caused by adenoviruses. As opposed to the older killed vaccine obtained from primary monkey kidney cells (see section 2.1), this preparation contains virulent adenoviruses in enteric-coated capsules for oral application. Delayed release of the live viruses by the pH-sensitive coating leads to a subclinical intestinal infection that induces immunity also against the more severe febrile respiratory disease [51]. Initially produced by Wyeth-Ayerst starting in the 1970s, production was stopped in 1996 for economic reasons [52]. The acute febrile adenovirus infections resurged, associated with significant medical costs and disruption of training schedules [52,53]. Teva Pharmaceuticals was contracted as a new manufacturer for production of the “Adenovirus Type 4 and Type 7 Vaccine, Live, Oral” on WI-38; this vaccine was approved by the FDA in 2011.

Another vaccine developed on WI-38 and still being produced on this cell line protects against rubella. The virus strain RA 27/3 (for example in MERUVAX II or as a one component in M-M-R II, both from Merck) was attenuated by Stanley Plotkin in the 1960s by serial passaging at successively lower temperatures, from 35 °C down to 30 °C [54,55,56].

Concern that the supply of finite cells derived from human embryos may eventually be limiting prompted research into alternative sources. To provide pre-tested diploid cells predicted to be susceptible to a wide range of human viruses, cultures from subhuman primates were established. One of the resulting cell lines, DBS-FRhL-2, was derived from the lung of a male rhesus fetus [57,58]. First signs of senescence appear in cultures after 60 cell doublings and further growth is arrested at a population doubling level (PDL) of approximately 74. Permissivity of DBS-FRhL-2 cells for poliovirus, Coxsackie virus A9, parainfluenza virus type 3, rhinovirus, vaccinia virus and rubella virus was shown to be comparable to control cell culture systems. A vaccine against severe gastroenteritis in infants and young children, Rotashield (Wyeth), was briefly produced on DBS-FRhL-2 cells until its (probably unwarranted) withdrawal in 1999 [59]; the cell bank deposit at the American Tissue Culture Collection (ATCC) is at passage level 10 [60].

### 2.4. Disadvantages and Advantages of Finite Cell Substrates

The paramount advantage of primary cells and finite cell lines for vaccine production is that an enormous amount of regulatory experience has accumulated in the decades since the 1930s. This advantage needs to be balanced against some disadvantages, with supply and costs being especially important.

Because of the finite lifespan of CEFs, embryonated eggs have to be harvested continuously, and each new preparation carries a certain risk of contamination with adventitious agents and variation in permissivity for the target virus. This property renders production an undesirably dynamic and open system with a complex logistics between the limited number of facilities that can supply such eggs and those that can process this substrate [61,62].

Supply with HDCs derived from characterized cryogenic seeds has also been a concern [63,64]. For this reason, the WHO initiated generation of a new MRC-5 seed bank. This bank originates from cells at a PDL of 7 and consists of 450 vials with PDL-12 cells expected to provide production substrate for at least 20 years [65].

Supply of cell substrate is not the only hurdle. It is also desirable to perform vaccine production in large suspension cell volumes in bioreactors. Furthermore, the preferred culture media are free of animal-derived components to reduce risk of contamination. Modern processes can increase yields, facilitate delivery of vaccines and reduce costs–very important parameters especially for vaccines intended for use in less affluent geographic regions (e.g., discussed in [66]). Adaptation of cells with finite number of population doublings and dependence on complex media and substrate anchorage to modern production processes appears not to be possible with the current technology. Continuous cell lines solve some of these limitations, but may introduce novel challenges.

## 3. Continuous Cell Lines

Continuous cell lines have been passaged beyond senescence. Although some properties, including karyotype and dependence on growth factors or substrate anchorage, may be in flux, the cells, in theory, have an unlimited life span. They have also a reduced host complexity, compared to the multiple cell lineages that may be present in tissue explants, so that variations in virus strain diversity and vaccine production yields can be reduced. This advantage was recognized already in 1953 in experiments where clonal L929 cells derived from a mouse fibrosarcoma were infected with herpes simplex and pseudorabies viruses [67]. At the same time, HeLa was studied for propagation of vaccine and field strains of poliovirus [68]. These experiments established that consistent production of human viruses should be possible without dependence on further primary material.

**Figure 1:**
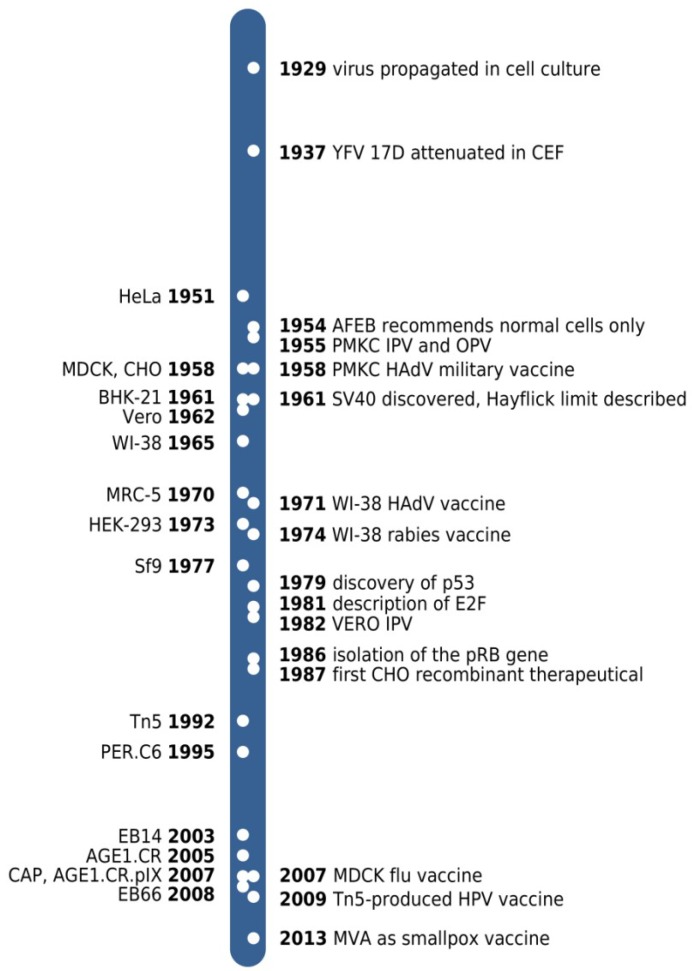
Time line of cell line derivations (left to the time bar) and key developments (right side) referred to in the text. YFV 17D, yellow fever virus 17D strain; CEF, chicken embryo fibroblasts; AFEB, American Forces Epidemiological Board; PMKC, primary monkey kidney cell; IPV and OPV, inactivated and oral polio vaccine, respectively; HAdV, human adenovirus; MVA, modified vaccinia virus Ankara.

### 3.1. Vero

The authors of the initial study, and others at the time [26], did not propose to produce human vaccines in a tumor-derived continuous cell line. The first continuous and aneuploid cell line that was used for human vaccine production (Figure 1) is the Vero cell line that originated from the kidney of an African green monkey (*Chlorocebus aethiops*) and was established by Yasumura and Kawakita in 1962 with the aim to facilitate investigation of the simian virus 40 (SV40, *Polyomaviridae*). Documentation and passage history of this cell line is exhaustive [60,69,70,71], from transfer at passage 93 (in 1964, from Chiba University in Japan to the laboratory of Tropical Virology, National Institute of Allergy and Infectious Diseases, Bethesda, MD, USA), cell bank submission at passage 113 (to the ATCC), cell bank generation at passage level 124 (resulting in the commercial product CCL-81), propagation to passage 129 for an additional cell bank (out of CCL-81, work performed at the Mérieux Institute (today Sanofi Pasteur)), and out of this cell bank to passage 134 for the 10–87 Vero Reference Cell Bank (RCB) of the WHO and to passage 137 for a series of working cell banks that includes a cell bank called LS-7. The LS-7 was used to produce polio and rabies vaccines [69] whereas ampoules from the 10–87 RCB are distributed for generation of further Master Cell Banks and Working Cell Banks. An additional cell bank out of CCL-81 is planned to obtain a source with lowest possible passage level for further distribution as the 10-87 RCB is nearing depletion [72].

Perhaps supported by a fortuitous defect in anti-viral signaling [73], the Vero cell line is permissive for a wide spectrum of viruses and is still in use for production of killed and live vaccines, including those against poliomyelitis (for example, PEDIARIX, Glaxo Smith Kline and IPOL, Sanofi Pasteur), rabies (VEROAB, Sanofi Pasteur), Japanese encephalitis (IXIARO, Intercell Biomedical), smallpox disease (ACAM2000, Sanofi Pasteur), and rotavirus gastroenteritis (Rotarix, Glaxo Smith Kline and RotaTeq, Merck).

### 3.2. MDCK

A substrate that has gained increasing importance is the MDCK cell line. Stewart Madin and Norman Darby established this continuous cell line from primary kidney cells of an apparently healthy adult cocker spaniel in 1958. The procedure for derivation and the first 49 passages appear not be published [74,75]. There also appears to be some confusion as to the sex of the donor animal that some publications list as female and others as male, and as to the actual strains that are being used in studies on epithelial junctions and cell polarity for which MDCK is especially suitable [74]. An unusual property of MDCK cells is a high permissivity for diverse strains of influenza A and B viruses [76]. There are also lineages of MDCK cells available that proliferate well in bioreactors in single-cell suspensions without dependence on microcarriers or calf serum. Because of this favorable combination of properties, MDCK-based production processes for killed vaccines against seasonal influenza have been developed. The first regulatory approval for a trivalent subunit vaccine against flu produced in MDCK cultures (OPTAFLU, Novartis) was granted in 2007 [77,78].

### 3.3. BHK, CHO and EB66

There are continuous cell lines that are currently not used for production of human vaccines but should not be omitted in this review. For example, the BHK21 cell line is an important host cell for vaccine research and production of animal vaccines, the CHO cell line has paved regulatory issues, and the EB66 cell line is an example for a modern approach in vaccinology.

The BHK21 cell line originates from the kidney explant of a 1 day-old Syrian hamster (*Mesocricetus auratus*) prepared in 1961 [79,80]. The aim of the experiments that led to this cell line was to study the mechanism of polyomavirus transformation. To test for soluble factors that may be involved in resistance to transformation, media were exchanged between treated and untreated cultures. On day 65 in culture, cells in the litter number 21 culture were observed to replicate rapidly although they had not “been knowingly exposed to polyoma virus during their propagation” [80] so that a spontaneous immortalization event appears to be the most plausible explanation. BHK cells are used for production of veterinary rabies vaccines. They are also highly permissive for a wide spectrum of viruses (including a virus host-restricted to avian cells [36]) and are frequently used in research on investigational vaccines (for example, [81,82]). However, no licensed human vaccine is being produced on this cell line.

Perhaps with the exception of the mysterious vesivirus isolate 2117 in bioreactor fluids [83], virology with the CHO cell line as host appears to be without surprises. As opposed to BHK, this cell line derived from an inbred (captive) female Chinese hamster (*Cricetulus griseus*) [84] is not permissive for many viruses [85,86,87]. However, interesting in the context of the regulatory aspects of vaccine production is that, in 1986, the first non-microbial recombinant protein licensed for use in humans was produced on CHO. The CHO cell line has a dynamic karyotype, can form tumors in nude mice and releases type C retroviral particles from endogenous proviruses [88,89,90]. The licensed protein, recombinant tissue-type plasminogen activator (rt-PA), effects thrombolysis in the emergency treatment of pulmonary embolism, myocardial infarction, and stroke. rt-PA is still in use today and an extremely efficacious recombinant therapeutical protein [91]. Points considered in the decision towards licensing may have included that t-PA requires processing for activity that cannot be provided by bacterial expression technology, that repeated administration of the microbial alternative (streptokinase) is associated with the risk of a neuropathic reaction, and that t-PA cannot be isolated in sufficient amount from human serum [89,92,93]. Furthermore, the use of human material for isolation of therapeutics was not a preferred option at a time where the AIDS pandemic was still unfolding [94]. Today, a diverse spectrum of therapeutic proteins [95] is being produced with this tumorigenic cell line that also harbors active endogenous retroviruses. The regulatory considerations and improvements in tracking of raw materials, cultivation of cells, product purification, and validated measures of virus inactivation that have advanced the recombinant protein field cross-pollinate also related processes for viral vaccine production.

EB66 (and CR.pIX, discussed below) is an avian continuous cell line. With such a cell line the dependence from embryonated eggs can be reduced and modern bioreactor processes in suspension are possible. The EB66 cell line originates from stem cells obtained from the egg yolk of Peking duck (*Anas* sp.) eggs in a massive development that involved 22,000 eggs [96,97,98]. No human vaccines appear yet licensed for production on this cell line but regulatory documentation appears to be complete for approval.

### 3.4. Insect Cell Lines

Insect cell lines are not directly a substrate for vaccine viruses but used as an expression system in combination with recombinant AcNPV (autographa californica multiple nucleopolyhedrovirus, *Baculoviridae*). This highly efficient expression system can also be used to generate virus-like particles (VLPs) that, due to their size and greater complexity compared to isolated antigens, can be more efficacious than conventional killed vaccines. Common insect cell lines are the Tn5 cell line (also called High Five or by its full designation BTI-TN-5B1-4) from the cabbage looper (*Trichoplusia ni*) [99] and the Sf9 cell line from the fall armyworm (*Spodoptera frugiperda*) [100].

Recently licensed vaccines out of the baculovirus expression system are Cervarix (Glaxo Smith Kline) against human papilloma virus (HPV, *Papillomaviridae*, produced on Tn5 cells; the other HPV vaccine, Gardasil (Merck), consists of VLPs produced in yeast) and Flublok (Protein Sciences) against seasonal influenza (purified hemagglutinin produced on Sf9 [101]).

### 3.5. Designed Cell Lines: Regulation of Cell Cycle Progression, Apoptosis and Senescence

Continuous cell lines develop out of primary cells after lesions occur in pathways that regulate senescence, apoptosis and cell cycle progression. Accidental lesions after prolonged passaging or induction with chemical mutagens can lead to spontaneously immortalized cell lines. If the lesions are induced by precise and targeted experimental manipulation the resulting cell line is a designed cell line. As discussed in greater detail below (see section 4.1), one advantage of designer cell lines is that the oncogenic risk associated with host cell-derived DNA, that may contaminate a vaccine, can be calculated with greater confidence.

Because some viruses have evolved factors that induce cell cycle progression to increase availability of biosynthetic compounds for the acute phase of infection, virology has contributed significantly to the level of knowledge about cell immortalization. One common biochemical motif of several viruses [102,103,104] is to disrupt the association of the negative regulator pRb [105] with E2F [106] transcription factors. The targets of E2F proteins include cyclins, cyclin dependent kinases (CDKs), and proteins important for DNA replication and repair. However, cell cycle progression out of the proper homeostatic or ontogenetic context may result in induction of senescence or apoptosis to interfere with tumor formation or further virus replication. Some of the previously liberated E2F members mediate this effect via activation of an alternate reading frame (ARF) in the INK4a/ARF gene locus. Increased levels of ARF lead to increased proteolysis of MDM2. This step allows increased activity of p53, a protein central to events that are important for induction of G1 cell cycle arrest and apoptosis [107]. Some viruses have therefore evolved factors that interfere with the regulation by p53 [104,108,109] to act in concert with the viral factor that attacks pRb.

Among the best known viral regulators of these cell cycle events are the large T antigen (LT) of SV40 [102,110,111,112,113,114], the E6 and E7 functional pair encoded by HPV [115], and the E1 genes of HAdV [103]. If such factors are expressed in a primary cell it may become immortalized.

Not all of these factors appear to be suitable for generation of a substrate intended for vaccine production. Some tumor viruses are associated with a greater degree of inducing malignancies than others, and any transfer of the more pleiotropic factors derived from such viruses to vaccine recipients should be avoided. For example, the high-risk HPV E6 protein not only disrupts p53 functions but also appears to modulate signaling complexes responsive to cell contact (which is important for metastasis), and may augment telomere maintenance (by itself a transforming activity as it impacts senescence) [104,116]. Furthermore, the E7 protein of high-risk HPV-16 interferes both with the regulated activity of histone deacetylases and histone acetyl transferases, and can directly (and independently of pRb) bind to and modulate activity of E2F1 and E2F6 [104]. HPV of the high risk groups has been associated with 99% of cervical cancer cases [115]. To establish this connection was instrumental for the successful efforts in development of a vaccine against cervical cancer [117] and awarded with a Nobel Prize for Harald zur Hausen in 2008.

Although the E1 proteins of HAdV serotype 2 and 5 can immortalize mammalian cells, these viruses are not associated with tumors in humans. One reason for this observation may be that E1A, the adenoviral factor responsible for liberation of E2F transcription factors, engages pRb in a complex together with Mdm2 in a way that leaves cytoplasmic p53 available for induction of mitochondrial outer membrane permeabilization [118]. Cell cycle progression induced by E1A therefore increases sensitivity to pro-apoptotic stimuli [103] and dependence on protective functions provided by two partially overlapping coding sequences in E1B. While E1B 55 K protein alleviates p53 accumulation in the nucleus, the E1B 19 K protein interferes with caspase activation that initiates in the mitochondrion [119].

In the context of vaccine substrates, the presence of adenovirus E1 genes appears to pose a minimal risk to vaccinees and yet allows targeted immortalization of certain primary cells.

### 3.6. Designed Cell Lines: HEK 293, PER.C6, CAP, CR.pIX

Directed immortalization of cells was initially investigated by infection with oncoviruses. However, such cell lines are not well suited as substrates for vaccines because the immortalizing factors should be continuously expressed to prevent that the cells progress to senescence or apoptosis [120]. This requires persistence of the oncovirus that may be accompanied by cytopathic effects [121,122,123,124], and increases the risks for contamination of a vaccine produced on such cell lines. The transformation observed in the HeLa cell line [125,126] was an exception because the genomic DNA of high-risk HPV 16 and 18 can become integrated into normal body cells in a way that can lead to unregulated expression of aggressive oncogenes with concomitant loss of infectious activity [127,128].

For the experimental immortalization of primary human cells with HAdV type 5, the infectious genomic DNA was therefore mechanically sheared prior to transfection to block generation of progeny virus [129,130]. The resulting HEK 293 cell line was later shown to have an adenoviral DNA fragment of 4.3 kb (less than12% of the tightly packed viral genome) that contains the E1 genes stably inserted into chromosome 19 [131].

The HEK 293 cell line is a valuable tool for investigation of E1-deleted replication defective adenovirus vectors. However, homologous recombination of some vector constructs with the integrated E1 region can lead to the rescue of replication competent adenoviruses that may impact safety of clinical application [132]. The PER.C6 cell line [133,134] is a truly designed cell line where immortalizing E1A and E1B are expressed from heterologous promoters and flanked by non-cognate sequences to minimize risk of recombination with adenoviral vectors. HEK 293 and PER.C6 are permissive for many vaccine viruses [135,136,137] and regulatory agencies are familiar with this approach of obtaining immortalized cell lines [138]. Both cell lines, HEK 293 and PER.C6, originate from aborted human fetuses. Additional E1-positive human cell lines (including the CAP cell line reported in 2007 [139]) have been derived from amniocytes obtained in the course of prenatal cytogenetic diagnosis [140,141].

Another advantage of transfecting immortalizing factors is that primary cell types can be targeted that normally are not permissive for a given virus. Mediated by E1 genes of the human adenovirus type 5, the avian cell line AGE1.CR from muscovy duck (*Cairina moschata*) was generated with the intention to replace chicken primary cells in vaccine production [142,143]. The further characterized CR.pIX cell line is a direct derivative of CR [144,145,146,147].

## 4. Regulatory Properties

Vaccines appear to be among the most efficacious and cost effective health interventions available [66,148]. However, they are usually given to healthy and sometimes very young recipients so that regulatory guidelines need to be strict. Furthermore, conventional vaccines protect individuals and populations but do not produce a visible recovery from disease similar to the role antibiotics can play in bacterial infections. For this reason, public attention can be biased towards adverse events.

The production substrates are considered an integral component of vaccine preparations and a large number of health regulatory guidelines, discussion papers and points to consider describe requirements for cell substrates. The remainder of this review will focus on the main concerns: risk of transforming factors that may co-purify with vaccines and potential contamination with pathogens.

### 4.1. Tumorigenicity

Tumorigenicity describes the capacity of viable cells to proliferate in a recipient. There appear to be no molecular markers for this property. Although chromosomal aberrations are hallmarks of many cancers they have also been observed in the non-tumorigenic MRC-5 cell line [50,149]. It therefore appears that stability of the karyotype of a cell line is not predictive [149,150] and tumorigenicity is therefore determined experimentally.

Assays for tumorigenicity include injection of viable cells into appropriate, usually immunosuppressed animals such as neonatal rats treated with anti-thymocyte serum or *Nu/Nu* genotype adult nude mice. If progressively growing nodules are observed, the level of tumorigenicity is determined by the time until nodules appear, the number of injected cells that is required to produce nodules, and whether nodules are detected distant from the injection site [150]. Tumorigenicity can differ between strains and passage levels of a cell line [75,151]. For example, Vero cells at the low passage level used for vaccine production are not tumorigenic but evolve the capacity to form progressing (and sometimes metastasizing) nodules in model animals at passage levels of 140 to 250, depending on prior cultivation intervals [69,151]. MDCK cells adapted to proliferation in suspension are highly tumorigenic (10 cells/immunosuppressed animal suffice for tumor formation) whereas MDCK cells that are still anchorage dependent are at an intermediate level (10^5^ cells can form nodules). As a reference, a cell line is weakly tumorigenic if 10^7^ cells/model animal are required for tumor induction (HEK 293 is such a cell line).

Regulatory discussions on tumorigenic cell lines for vaccine production are evolving. For example, in 1954, the American Forces Epidemiological Board (AFEB) had to decide on new substrates for production of a vaccine against acute respiratory disease caused by adenoviruses. They specified that live and killed vaccines should be produced only on “normal” cells to avoid that vaccine recipients may be exposed to cells or factors with the capacity to induce tumors [152,153,154]. Today it is known that the human diploid cell line WI-38 also is not tumorigenic. But in 1954, the strict definition of the AFEB excluded these cell lines because they are sub-passaged and therefore not considered to be “normal” anymore. Discovery of adventitious agents in the approved primary monkey kidney cells (see section 4.3) has contributed to consideration of the pre-tested and well characterized human finite cultures for vaccine production [153]. Starting in 1963, oral adenovirus vaccines produced on WI-38 were tested on army recruits and, starting in 1971, used routinely to control acute respiratory disease [51,53].

Risk-benefit considerations change with biotechnological advances and positive experiences with recombinant protein therapeutics. Cytopathic effect and vaccine production processes are expected to be highly effective in removing all viable cells from vaccine preparations. With appropriate characterization, tumorigenic cell lines such as MDCK for production of influenza vaccines and PER.C6 for vectored vaccines are therefore now acceptable [150].

### 4.2. Oncogenicity

Oncogenicity describes the capacity of cellular components within the host cell to induce tumors in a recipient. The most important component potentially associated with transforming activity is host-cell derived DNA that may express or activate oncogenes. Ribonucleoprotein complexes and other entities commonly found in cell lysates perhaps cannot persist long enough to support malignant transformation that is postulated to proceed in several stages [155]. Replication-competent oncoviruses (such as SV40 in primary monkey kidney cultures) that can be transferred not only as virions but also as infectious genomes are discussed below in the context of adventitious agents.

A maximum admissible amount of host cell-derived DNA in oral and intranasal vaccines has not been defined but final formulations should be discussed with authorities [150]. There are also no levels defined for vaccines produced on diploid or primary cell substrate. However, a parenteral vaccine produced on a continuous cell line may contain no more than 10 ng of host cell-derived DNA per dose [156]. This value is the result of considerations that estimate the probability for an oncogene to be encoded and successfully transferred with a given segment of co-purified DNA. Enzymatic fragmentation or treatment with β-propiolactone may further reduce any risk associated with contaminating DNA [157] but some processing steps cannot be performed without loosing potency of live vaccines.

The current value of 10 ng of substrate DNA per dose of parenteral vaccine is expected to overestimate the risk of transferring oncogenic activity. For example, a much higher exposure is experienced in blood transfusions [158], human cells appear not to incorporate DNA provided by intramuscular injection as efficiently as the rodent test animals [159], and no tumors were observed in a longitudinal study with monkeys that were injected with up to 1000 µg of DNA derived from the continuous human PER.C6 cell line [160]. The risk considerations may be further refined and facilitated if the nature of the immortalizing factors present in the continuous cell line are known. Some advantages have been summarized recently for designed cell lines obtained with adenovirus E1 proteins [161].

### 4.3. Adventitious Agents

Adventitious agents are potentially infectious entities unrelated to the vaccine strain that may copurify with a final preparation. They are a major concern in processes with primary material [61] and continuous cell lines, both of invertebrate [162] and vertebrate [83] origin. Introduction can occur from exogenous sources (such as hepatitis B virus-contaminated human serum used in the 17D vaccine formulation [163]) or from an inapparent infection of the cell substrate.

Contamination of vaccines against poliomyelitis and adenoviruses between 1955 and 1963 with SV40 is an early example of a serious adverse event originating from the cell substrate. SV40 is a natural pathogen of Asian macaques and was present in the primary monkey explants used for vaccine production [164,165,166]. Estimates on exposure to contaminated vaccine range between 10 and almost 100 million children and thousands of army recruits in the USA. Interpretation is confounded because SV40 may have not been completely inactivated by the treatment with formalin used to prepare killed vaccines [166], because more than one strain of SV40 may have been carried into vaccines [167,168] and because tainted vaccines may have been administered in Eastern Europe until the 1980s [166].

SV40 can induce tumors in laboratory animals but there still is a controversial discussion on whether SV40 infection can also induce tumors in humans [164,165,166,168,169,170]. Epidemiology does not suggest that tumor incidence has increased with SV40 exposure but confirmation with molecular markers is not reliable: antibodies induced by human polyomavirus cross-react with SV40 and not all of those who are exposed to SV40 (as determined by virus in the stool) seroconvert.

Another adventitious agent was reported in 1967 with the first isolation of Marburg virus (MARV, *Filoviridae*) out of serum samples of patients submitted to hospitals with serious disease. The patients had processed monkey tissues and kidney cultures intended for vaccine production [171]. Out of 32 patients in three clusters 7 died. Because of this incident, quarantine regimes that allow 30 days for observation of imported non-human primates were implemented. In 1989, in one such unit (Hazelton Research Products, Inc, Reston, VA, USA; today Covance Inc.), symptoms of severe disease were observed in several cynomolgus monkeys (*Macaca fascicularis*) originating from an animal breeding facility in the Philippines. Simian hemorrhagic fever (SHF) was suspected and samples sent to the U.S. Army Medical Research Institute of Infectious Diseases for further diagnostics [172]. SHF is caused by a (+)-strand RNA virus (SHFV) of the *Arteriviridae* that is not known to be dangerous for humans. Surprisingly, in addition to SHFV, some animals also appeared to have been infected with a relative of the Ebola virus. This first of the *Filoviridae* to be isolated from a non-African source today is known as Reston virus (RESTV, previously REBOV) [173]. Additional incidents with RESTV-infected cynomolgus monkeys imported from the Philippines were reported in 1992 and 1996 [174]. The virus strain from the Philippines may be endemic in domestic pigs and appears not to be virulent for humans [175,176].

Increasing burden on monkey populations required for scientific purposes [18,177] and increasing recognition of the dangers associated with adventitious agents in primary monkey cells [178] had to be balanced with risks associated with heteroploidy as a result of serial propagation of finite cultures. In 1972, the human diploid cell line WI-38 was approved for manufacture of live polio vaccine [153].

Under exceptional circumstances adventitious agents may be tolerated in a vaccine. For example, released vaccines produced with material from embryonated chicken eggs were shown to contain retroviral particles [179,180]. The particles are derived from endogenous retroviruses, proviruses that are vertically transmitted and can account for up to 3% of the chicken genome and 8% of the human genome [181,182,183,184]. The majority of the human endogenous retroviruses are defective but in chickens they are capable of forming particles that contain reverse transcriptase activity. In 1998, the WHO recommended that production of the extremely beneficial vaccines on chicken cells should continue because there have been no cases of human disease due to the chicken-derived particles and because alternative substrates with an otherwise similar degree of proven safety are not yet available [185]. Continuous cell lines from chicken appear not to provide a true alternative. For example, the EB14 cell line [186] intended for vaccine production was not investigated further because of the presence of endogenous avian retroviruses [97]. Certain waterfowl species appear not to shed such particles and continuous duck cell lines have been generated to avoid this problem [97,143].

Another contamination that appears not to pose a risk for human recipients, as opposed to the severe gastroenteritis against which the vaccine protects, are porcine circoviruses (PCV1 and PCV2; *Circoviridae*) in the human rotavirus vaccine [187,188]. PCV1 and PCV2 are globally ubiquitous and probably were introduced together with porcine pancreatic trypsin used for cultivation of the Vero production substrate.

The advantage of processing non-primary substrates is that testing for adventitious agents can be performed on cell banks ahead of production. Suggested assays [150,189] screen cell lysates for specific pathogens by PCR or antibody induction after inoculation in suitable model animals. Transmission electron microscopy is used to investigate cells for known and previously uncharacterized agents. Co-cultivation with a panel of indicator cell lines should expose viruses via signs of cytopathic effect. Highly sensitive product-enhanced reverse transcriptase assays are directed against retrovirus contamination. Inoculation of lysates into suckling and adult mice, guinea pigs and embryonated eggs screens for virulent pathogens that may be associated with latent infection in cell cultures. Chemical induction and co-cultivation with sentinel cells can be included to reveal occult infections [190].

To investigate virus preparations being obtained on a given host cell, future assays may include next generation sequencing (NGS) for confirmation of molecular markers that may be predictive for vaccine properties [191]. The impact of the host cell or production environment can be beneficial and lead to attenuation (as has been shown for YFV 17D, MVA and the measles virus vaccine strain Edmonston, all obtained by passage on galline cells [29,30,192], attenuated Japanese encephalitis SA 14 viruses that were propagated on primary hamster kidney cells [193,194], or RA 27/3 derived from a human diploid cell line [56]). Serial passaging of a virus on certain cell lines can also lead to undesired properties, including a surprising loss of reactogenicity as has been shown for an investigational vaccine against dengue fever [195]. However, NGS may also yield a plethora of unrelated viral and microbial sequences so that significance for vaccine development and production may have to be interpreted with care.

### 4.4. Public Concerns and Misconceptions

There are misconceptions about vaccines that may interfere with acceptance and can have severe consequences, for example because global efforts to control an infectious disease are disrupted or because certain groups are rendered especially vulnerable to some diseases [196,197]. Some misconceptions concern the production substrates. For example, the “OPV/AIDS theory” proposes that vaccine trials in Africa have introduced an ancestral HIV-1 into the human population. According to this theory, some of the 1957–1960 oral polio vaccine (OPV) may have been produced in chimpanzee primary cells. It is further suggested that this additional substrate may have contaminated vaccine preparations with primate immunodeficiency virus SIVcpz that may have evolved into group M human immunodeficiency virus [198]. This iatrogenic origin of AIDS appears to be unlikely. A recount of the OPV trials, molecular phylogenetic analysis, and sequence data in archival vaccine stocks strongly point to multiple independent zoonotic transmissions decades prior to the OPV trials [199,200,201,202,203].

Another aspect regarding vaccine acceptance and production substrates relates to processing. Although different rules may apply for medication as opposed to food, some religious communities may decline a vaccine out of fear that porcine excipients are present [204]. For example, porcine trypsin is used to resuspend adherently growing cells or to activate some viruses for replication. Such concerns may be mitigated by replacement with recombinant alternatives and information on the high degree of depletion of the offending substances in the final product [204].

Cause for religious and ethical concern is also that human diploid and some designed cell lines are derived from tissues of human fetuses, and that vaccination may constitute an illicit cooperation with abortion. In one essay it is suggested that research and development should continue only without “unethical cell strains” [205]. However, other detailed contemplations have come to the conclusion that parents should not risk a child’s health because of an abortion in the past [206]. Most importantly, there appears to be no causal link between benefit from vaccination and the act of abortion (hence, no illicit cooperation) because the fetus was not killed with the intention to develop a cell line or to isolate a vaccine strain [54,204]. In careful considerations, representatives from different religious groups come to the conclusion that the application of vaccines is in agreement with the principle that life should be protected and harm be prevented [204].

## 5. Conclusions

Cell lines are an integral component of vaccine research, development and production. It is therefore not surprising that aspects in the history of vaccinology can be traced through the history of derivation and investigation of cell lines. Consequently, a wide spectrum of cell substrates is available, from primary to continuous cell cultures, from mammalian to avian and even invertebrate animals.

One advantage of primary chicken cells and embryonated chicken eggs is experience that has its roots in the 1930s. Advantages of novel cell substrates include process options for production at large scales in chemically defined media. Future developments may focus on improved packaging and helper cell lines for generation of host-restricted, highly attenuated viral vectors directed against certain cancers and chronic infectious diseases [207,208].
